# The SNP rs1883832 in CD40 Gene and Risk of Atherosclerosis in Chinese Population: A Meta-Analysis

**DOI:** 10.1371/journal.pone.0097289

**Published:** 2014-05-14

**Authors:** Yan Yun, Chi Ma, XiaoChun Ma

**Affiliations:** School of Medicine, Shandong University, Ji’nan, Shandong, People’s Republic of China; Hospital Italiano de Buenos Aires, Argentina

## Abstract

**Background:**

The complications of atherosclerosis such as coronary and cerebrovascular disease, are the most prevalent causes of mortality and morbidity worldwide. A single nucleotide polymorphism (SNP) rs1883832 (-1C/T) in CD40 gene has been recently suggested to contribute to the susceptibility to atherosclerosis in Chinese population; however, previous genetic association studies yielded inconsistent results.

**Methods:**

A meta-analysis of eligible studies reporting the association between rs1883832 and atherosclerosis in Chinese population was carried out.

**Results:**

Pooling 7 eligible case-control studies involving 2129 patients and 1895 controls demonstrated a significant association between rs1883832 and atherosclerosis under dominant model [odds ratio (OR) = 1.631, 95% confidence interval [CI] [1.176, 2.260] in Chinese population with evident heterogeneity. Meta-regression analysis indicated that the heterogeneity could be completely explained by disease category. In subgroup analysis, rs1883832 conferred ORs of 2.866 (C/C versus T/T, 95%CI [2.203, 3.729]) and 1.680 (C/T versus T/T, 95%CI [1.352, 2.086]) for coronary artery disease (CAD) under co-dominant model without heterogeneity. Similar results were obtained for acute coronary syndrome (ACS) (C/C versus T/T, 3.674, 95%CI [2.638, 5.116]; C/T versus T/T, 1.981, 95%CI [1.483, 2.646]). The other genetic models including dominant, recessive and additive models, yielded consistent results without heterogeneity for CAD and ACS, respectively. However, a protective role was found for C allele in ischemic stroke (IS) under recessive model (0.582, 95%CI [0.393, 0.864]) and additive model (0.785, 95%CI [0.679, 0.909]) with reduced heterogeneity.

**Conclusions:**

This meta-analysis provided evidence of association of rs1883832 C allele with an overall increased risk of atherosclerosis but distinct effect of C allele on CAD (including ACS) and IS in Chinese population, respectively.

## Introduction

Atherosclerosis, with clinical consequences including coronary artery disease (CAD) and ischemic stroke (IS), is one of the leading causes of death worldwide [1]. Accumulating evidence has suggested a pivotal role of CD40-CD40L interaction in the pathogenesis of atherosclerosis [2], [3]. CD40, a 50-kDa cell surface transmembrane glycoprotein receptor of the tumor necrosis factor receptor (TNFR) superfamily, which is expressed on the surface of immune cells as well as non-immune cells, determines T cell responses to antigen presentation and B cells immunoglobulin isotype switching [4]. A C/T single nucleotide polymorphism (SNP) (rs1883832) in the 5′ untranslated region of CD40 gene located at the -1 position within the Kozak sequence has been recently associated with CD40 protein expression and susceptibility to atherosclerotic diseases in Chinese population [5]–[7]. However, consistent results were not obtained from previous genetic association studies.

We undertook the present meta-analysis of relevant literature to evaluate the association of rs1883832 (-1C/T) in CD40 gene with atherosclerosis (overall, CAD, acute coronary syndrome [ACS] and IS) in Chinese population, with the following objectives: A) to estimate C allele frequencies in controls; B) to assess if the effect of rs1883832 on atherosclerosis (overall, CAD, ACS and IS, respectively) is statistically significant in Chinese population; if so, C) to estimate the magnitude of effect of C allele and determine the most appropriate genetic model (s) for atherosclerosis (overall, CAD, ACS and IS, respectively) in Chinese population.

## Materials and Methods

### Eligibility Criteria

We sought eligible studies which met the following criteria: (1) published studies based on case-control design assessing the association between rs1883832 (CD40 gene -1C/T) and atherosclerosis; (2) involving subjects restricted to Chinese ethnicity; (3) providing sufficient data for calculating genotypic odds ratio (OR) with corresponding 95% confidence interval (95% CI). Non-English language studies were excluded, as were review articles, case reports, editorial comments and animal studies. It inevitably introduces a source of bias when a meta-analysis incorporates the genetic association studies from the same or different research group(s) utilizing duplicate samples. Thereby, it is obligatory to exclude in advance those repetitive publications to avoid the duplicate-study effects on our meta-analysis. The recruiting periods and hospitals were checked thoroughly for all the included studies. Additionally, the corresponding authors were contacted directly via email to confirm whether or not there were duplicate results to be eliminated.

### Search Strategy

MEDLINE and EMBASE were independently searched by two reviewers (Xiaochun Ma and Chi Ma) for potentially eligible studies from the earliest available date to August 2013. The search terms used either alone or in combination included “rs1883832-1C/T, CD40, TNFRSF5, atherosclerosis, coronary artery disease, acute coronary syndrome, ischemic stroke and cerebral infarction”. Reference lists from retrieved articles were also manually searched for articles meeting our criteria. Abstracts identified using our search strategies were reviewed separately by two reviewers (Xiaochun Ma and Yan Yun). The full-text articles that potentially met criteria were then reviewed in duplicate to determine the inclusion in the analysis. Then all the authors further critically reviewed the studies upon which the 2 reviewers disagreed. Either the inclusion or exclusion of a certain study was agreed upon by all the authors finally.

### Data Extraction

Data extraction was performed independently by two reviewers (Xiaochun Ma and Chi Ma). In the process of data abstraction, the two authors were blinded to the information of each research group conducting the study. Extracted data included: (1) author’s name, year of publication and journal; and (2) study design, sample size, characteristics of cases and controls (geographic location or ethnicity of study population, control source, male/female rate and mean age), genotyping method, distribution of genotypes and alleles in cases and controls. The quality of studies was assessed with the Newcastle-Ottawa scale (NOS) for observational studies [8]. Eight items, in each of which a series of response options is provided, are categorized into three perspectives in NOS: four for the Selection of the study group (S), one for the Comparability (C) of the groups and three for Exposure of interest (E). A ‘star system’ is utilized for the assessment of study quality, such that a study can be awarded a maximum of one star for each numbered item within the Selection and Exposure categories while a maximum of two stars can be assigned for Comparability. The NOS scores from zero to nine stars [9].

### Statistical Analysis

Hardy-Weinberg equilibrium was assessed again for each study by goodness-of-fit x^2^ test (P>0.05) only in control groups. Pooled frequency of the A allele was estimated using the inverse variance method previously described by Thakkinstian et al. [10] in which random-effects or fixed-effects model was applied, depending on whether or not there is evident heterogeneity for allele frequencies across studies. Random-effects model introduces a ‘weight term’ that accounts for between-study variation and weight adjustment. Three crude ORs with corresponding 95% CIs, i.e. crude OR1 (C/C versus T/T), crude OR2 (C/T versus T/T) and crude OR3 (C/C versus C/T) were calculated for rs1883832. Cochran’s Q statistic and the I^2^ metric for heterogeneity were also estimated separately for crude OR1, OR2 and OR3. Three modified ORs and various genetic models would be measured afterwards only if there was no evident heterogeneity. For the Q statistic, a p value of less than 0.1 was considered statistically significant. For the I^2^ metric, I^2^>50% indicates significant heterogeneity [11]. If heterogeneity was evident for at least one of the 3 crude ORs, a meta-regression model was employed to explore possible sources of heterogeneity across studies. Subgroup analysis was then conducted on the basis of the results of meta-regression analysis. If there was no heterogeneity, logistic regression analysis was performed for determining the gene effect, which protected against inflated type I error in the context of multiple tests. The overall gene effect was evaluated using likelihood ratio (LR) test. If the main effect of the genotype was statistically significant, three modified ORs, i.e. OR1 (C/C versus T/T), OR2 (C/T versus T/T), and OR3 (C/C versus C/T) were further calculated using logistic regression model. Three pairs of crude and modified ORs were then utilized to suggest the most appropriate or ideal genetic model previously described by Thakkinstian et al. [10], [12] as follows:

If OR1 = OR3≠1 and OR2 = 1, then a recessive model is suggested.

If OR1 = OR2≠1 and OR3 = 1, then a dominant model is suggested.

If OR1>OR2>1 and OR1>OR3>1 (or OR1<OR2<1 and OR1<OR3<1), then a co-dominant model is suggested.

Besides the finest genetic model, several other possible genetic models were also assessed: co-dominant model (modified OR1 and OR2), (C/C versus T/T) and (C/T versus T/T); dominant model (OR4), (C/C and C/T) versus T/T; recessive model, C/C versus (C/T and T/T) (OR5); additive model (OR6) (table S1). For additive model, scores of 0, 1, and 2 were assigned to genotype CC, CT, and TT respectively and per-allele ORs were calculated by logistic regression model. Sensitivity analysis was performed to assess the effects of each study (especially the studies which do not observe HWE), on the pooled results. Publication bias was assessed using the Funnel plot method with Egger’s test. Random-effects models were chosen for pooling data and a p value of less than 0.05 was considered statistically significant for all estimates except for Q tests of heterogeneity. Agreement assessment between reviewers was evaluated using the Cohen k statistic. Stata 12.0 and Reviewer Manager 5.1 were utilized for performing the meta-analysis.

## Results

### Eligible Studies

**Figure 1:**
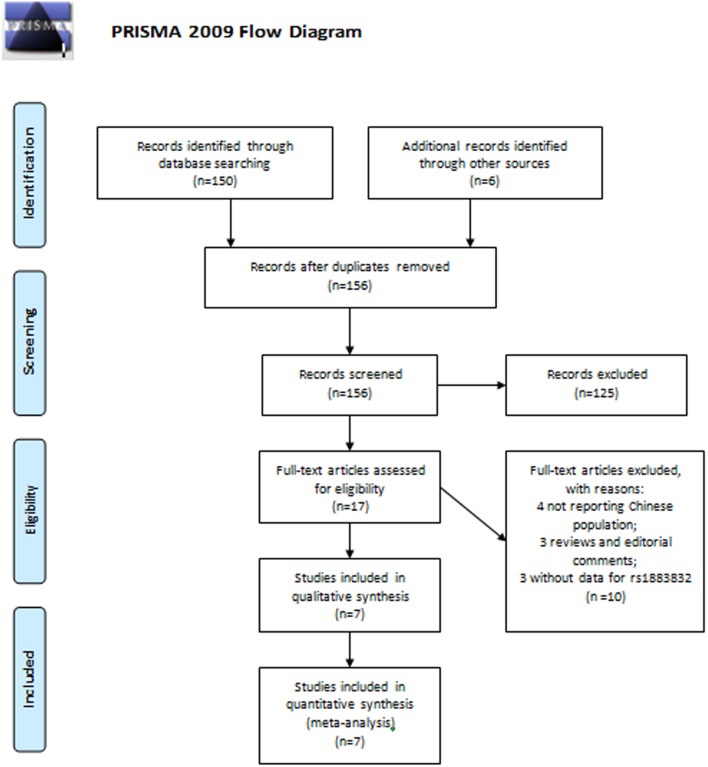
The PRISMA recommended flow-diagram depicting the methodology of article selection for this meta-analysis.

A total of 156 potential eligible citations were identified with the literature search, among which there were no articles written and published in languages other than English and Chinese. The titles of these citations were reviewed and 125 were rejected leaving 31 potentially eligible studies. After review of abstracts, an additional 14 were rejected. Seventeen studies were then retrieved in full-text to determine the inclusion. Assessment agreement of study selection between the two reviewers led to a k score of 0.84 (95% CI [0.79, 0.89]). Then a further discussion was conducted among all the authors with regard to the studies upon which the 2 reviewers disagreed. Finally, corporate critical review of full-text excluded 4 studies for not reporting Chinese population, 3 studies for being reviews and editorial comments, 3 studies for without data for rs1883832 (figure 1). All the included studies in this meta-analysis were written and published in English. Furthermore, there is currently no genetic association study concerning rs1883832 in atherosclerosis in other ethnicities, based on our search result.

### Study Characteristics

Ultimately, 7 studies (5 on coronary artery disease [CAD] and 2 on ischemic stroke [IS]) involving a total of 4024 subjects (2129 patients and 1895 controls) met our criteria and were included in this meta-analytical study [6], [7], [13]–[17]. The corresponding authors of our included studies were contacted and they confirmed that there were no duplicate results utilized in multiple studies to be deleted. Characteristics of the included studies are detailed in table 1. The distributions of genotypes and alleles in the individual studies for rs1883832 were shown in table 2. Within 7 included studies, one study was inconsistent with HWE [14]. All the studies we included gained a score of S3C1E3 based on the Newcastle-Ottawa scale.

**Table 1:** The characteristics of the studies included in the meta-analysis for the association of rs1883832 with atherosclerosis.

First author,Published year	Ethnicity	Controlsource	Studytype	Genotypingmethod	Diseasecategory	Case/Control	Male/female ratio		Mean age	
							Case	Control	Case	Control
Li Y, 2007*	Chinese	Population based	Replication	PCR-RFLP	ACS	160/92	3.57	3.60	64.0	59.0
Wang CP, 2009*	Chinese	Patients angiographicallynormal	Replication	PCR-RFLP	CAD	474/225	2.04	2.08	63.5	62.4
Tian CX, 2010*	Chinese	Population based	Replication	PCR-RFLP	ACS	248/206	1.76	1.94	63.8	59.8
Yan JC, 2010*	Chinese	Population based	Replication	PCR-RFLP	ACS,SA	399/163	3.53	3.41	63.8	62.8
Wang M,2011*	Chinese	Population based	Replication	PCR-RFLP	ACS	160/180	3.57	3.62	64.4	60.7
Ma Y, 2013^#^	Chinese Han	Population based	Replication	PCR-RFLP	IS	286/336	1.55	1.20	66.1	67.5
Zhang BK,2013^#^	Chinese Han	Population based	Replication	PCR-RFLP	CI	402/693	2.14	1.14	63.2	50.5

Abbreviation: PCR-RFLP, polymerase chain reaction-restriction fragment length polymorphism; ACS, acute coronary syndrome; CAD coronary artery disease;

SA, stable angina; IS, ischemic stroke; CI, cerebral infarction.

Symbols: ‘*’ indicates the study on coronary artery disease; ‘^#^’ indicates the study on ischemic disease.

**Table 2:** Distribution of genotypes and alleles in the individual study.

First author,published year	Genotype							Gene allele				Allele frequency		HWE for controls	
	Case			Control				Case		Control		Case	Control		
	CC	CT	TT	CC	CT	TT	Total	C	T	C	T	C	C	x2	P
Li Y,2007	45	104	11	12	66	14	252	194	126	90	94	0.61	0.49	17.45	2.956E-05
WangCP, 2009	121	257	96	35	118	72	699	499	449	188	262	0.53	0.42	1.37	0.242
TianCX, 2010	96	116	36	42	102	62	454	308	188	186	226	0.62	0.45	1.6788E-05	0.997
Yan JC,2010	95	216	88	26	87	50	562	406	392	139	187	0.51	0.43	1.35	0.245
WangM,2011	53	87	20	41	96	43	340	193	127	178	182	0.60	0.49	0.80	0.370
Ma Y,2013	54	196	36	112	173	51	622	304	268	397	275	0.53	0.59	1.41	0.234
ZhangBK,2013	110	216	76	243	335	115	1095	436	368	821	565	0.54	0.59	6.38E-04	0.980

Abbreviation: HWE, Hardy-Weinberg equilibrium.

### Pooled Frequency of C allele in Control Groups

The pooled frequency of the C allele in Chinese controls was 46.8% (95% CI [41.1%–52.5%]) under random-effects model. Heterogeneity was statistically significant (p heterogeneity<0.001, I^2^ = 91.5%) (table 3). When pooled separately, the frequency of the C allele in the studies on CAD was 45.1% (95% CI 42.7%–47.4%) under fixed-effects model without evident heterogeneity (p heterogeneity = 0.156, I^2^ = 39.7%) (table 4) while the frequency in the studies on IS was 49.9% (95% CI 31.9%–67.8%) under random-effects model with extreme heterogeneity (p heterogeneity<0.001, I^2^ = 98.4%) (table 5).

**Table 3:** Meta-analysis for the association of rs1883832 with atherosclerosis.

Genetic model	OR 95% CI	P	P heterogeneity	I^2^
C/C versus T/T	Crude OR1:1.926 (1.044, 3.555)	0.036	<0.001	88.5%
	Modified OR1:1.896 (1.183, 3.041)	0.008	–	–
C/T versus T/T	Crude OR2:1.502 (1.212, 1.862)	<0.001	0.183	32.1%
	Modified OR2:1.474 (1.239, 1.753)	<0.001	–	–
C/C versus C/T	Crude OR3:1.214 (0.758, 1.942)	0.42	<0.001	87.7%
	ModifiedOR3: 1.255(0.841, 1.873)	0.265	–	–
Dominantmodel (C/C and C/T)versus T/T	OR4: 1.631(1.176, 2.260)	0.003	0.001	72.5%
Recessive modelC/C versus (C/Tand T/T)	OR5: 1.370(0.821, 2.287)	0.228	<0.001	90.5%
Additive model	OR6: 1.361(0.981, 1.889)	0.065	<0.001	90.4%

Included publications: CAD: Li Y, 2007; Wang CP, 2009; Tian CX, 2010; Yan JC, 2010; Wang M, 2011; IS or CI:

Ma Y, 2013; Zhang BK, 2013.

Likelihood ratio test: LR = 23.82, P<0.001.

Pooled C allele frequency: 46.8% (41.1%–52.5%), P heterogeneity<0.001, I^2^ = 91.5%.

Abbreviation: ACS, acute coronary syndrome; CAD coronary artery disease; IS, ischemic stroke; CI, cerebral infarction;

OR, odds ratio; 95% CI, 95% confidence interval.

**Table 4:** Meta-analysis for the association of rs1883832 with coronary artery disease.

Genetic model	OR 95%CI	P	P heterogeneity	I^2^
C/C versus T/T	Crude OR1: 2.869 (2.197, 3.746)	<0.001	0.444	0%
	Modified OR1: 2.866 (2.203, 3.729)	<0.001	–	–
C/T versus T/T	Crude OR2: 1.691 (1.359, 2.105)	<0.001	0.838	0%
	Modified OR2: 1.680 (1.352, 2.086)	<0.001	–	–
C/C versus C/T	Crude OR3: 1.688 (1.352, 2.109)	<0.001	0.684	0%
	Modified OR3: 1.707 (1.366, 2.132)	<0.001	–	–
Dominant model (C/C and C/T) versus T/T	OR4: 1.965 (1.594, 2.423)	<0.001	0.58	0%
Recessive model C/C versus (C/T and T/T)	OR5: 1.971 (1.594, 2.438)	<0.001	0.603	0%
Additive model	OR6: 1.689 (1.480, 1.927)	<0.001	0.403	0.6%

Included publications: Li Y, 2007; Wang CP, 2009; Tian CX, 2010; Yan JC, 2010; Wang M, 2011.

Likelihood ratio test: LR = 63.73, P<0.001.

Pooled C allele frequency: 45.1% (42.7%–47.4%), P heterogeneity = 0.156, I^2^ = 39.7%.

Abbreviation: ACS, acute coronary syndrome; CAD coronary artery disease; IS, ischemic stroke; CI, cerebral infarction;

OR, odds ratio; 95% CI, 95% confidence interval.

**Table 5:** Meta-analysis for the association of rs1883832 with ischemic stroke.

Genetic model	OR 95%CI	P	P heterogeneity	I^2^
C/C versus T/T	Crude OR1: 0.684 (0.506, 0.926)	0.014	0.933	0%
	Modified OR1: 0.666 (0.477, 0.930)	0.017	–	–
C/T versus T/T	Crude OR2: 1.216 (0.749, 1.974)	0.429	0.093	64.6%
	Modified OR2: 1.190 (0.866, 1.635)	0.283	–	–
C/C versus C/T	Crude OR3: 0.556 (0.341, 0.907)	0.019	0.04	76.4%
	Modified OR3: 0.562 (0.402, 0.787)	0.001	–	–
Dominant model (C/C and C/T) versus T/T	OR4: 0.992 (0.691, 1.423)	0.965	0.188	42.3%
Recessive model C/C versus (C/T and T/T)	OR5: 0.582 (0.393, 0.864)	0.007	0.085	66.3%
Additive model	OR6: 0.785 (0.679, 0.909)	0.001	0.550	0%

Included publications: Ma Y, 2013; Zhang BK,2013.

Likelihood ratio test: LR = 22.04, P<0.001.

Pooled C allele frequency: 49.9% (31.9%–67.8%), P heterogeneity<0.001, I^2^ = 98.4%.

Abbreviation: IS, ischemic stroke; OR, odds ratio; 95%CI, 95% confidence interval.

### Meta-analysis of Association between rs1883832 and Atherosclerosis

**Figure 2:**
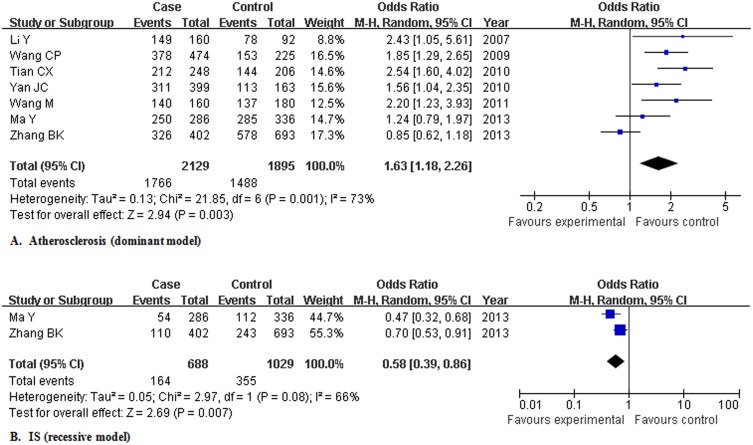
Meta-analysis for the association of rs1883832 with atherosclerosis and ischemic stroke in Chinese population. Abbreviation: IS, ischemic stroke; M-H, Mantel-Haenszel method.

For atherosclerosis, the 7 studies (n = 4024, 2129 patients and 1895 controls) were pooled for three crude ORs and Cochran’s Q statistic and the I^2^ metric indicated heterogeneity for OR1 and OR3 but not for OR2. To explore potential sources of between-study heterogeneity, a meta-regression model was employed using the data for crude OR1. After initially running an empty regression [18] (in which no variable was introduced) to evaluate the baseline value for tau^2^ (tau^2^ = 0.5276), a univariate model was then performed by adding disease category (CAD and IS), which reduced the tau^2^ value to 0 (adjusted R^2^ value 100%, p = 0.001). It indicated that the heterogeneity could be totally explained by category of atherosclerotic diseases. Pooling all the 7 studies yielded crude OR1 of 1.926 (1.044, 3.555), OR2 of 1.502 (1.212, 1.862) and OR3 of 1.214 (0.758, 1.942). Logistic regression showed that the overall gene effect was statistically significant, with the modified OR1, OR2, and OR3 being 1.896 (1.183, 3.041), 1.474 (1.239, 1.753), and 1.255 (0.841, 1.873), respectively. Those results suggested a dominant model of gene effect on atherosclerosis (OR4 1.631, 95% CI [1.176, 2.260]) (figure 2A). Several other possible genetic models were also estimated: co-dominant model, OR1 and OR2 being 1.896 (1.183, 3.041) and 1.474 (1.239, 1.753); recessive model, OR5 being 1.370 (0.821, 2.287); additive model, OR6 being 1.361 (0.981, 1.889). All the potential genetic models showed evidence of significant heterogeneity (table 3). Subgroup analysis by disease category was thus further performed for CAD, IS and ACS, respectively.

**Figure 3:**
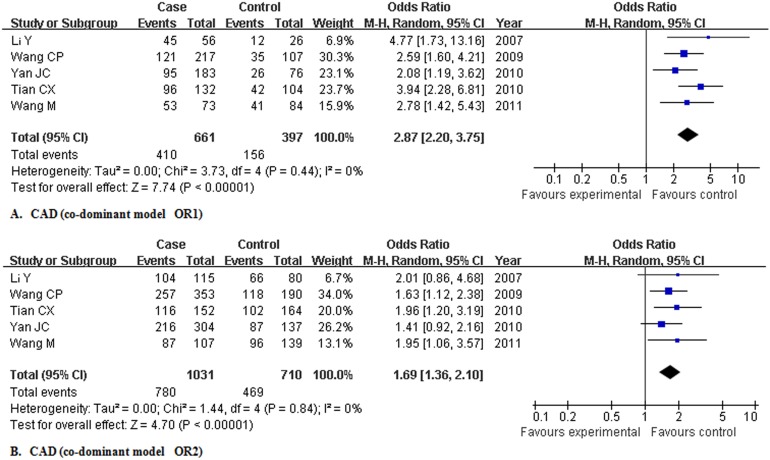
Meta-analysis for the association of rs1883832 with coronary artery disease in Chinese population. Abbreviation: CAD, coronary artery disease; OR, odds ratio; M-H, Mantel-Haenszel method.

For CAD, in subgroup analysis, pooling the 5 studies (n = 2307, 1441 patients and 866 controls) yielded crude OR1 of 2.869 (2.197, 3.746), OR2 of 1.691 (1.359, 2.105) and OR3 of 1.688 (1.352, 2.109) without heterogeneity. Logistic regression showed that the overall gene effect was statistically significant, with the modified OR1, OR2, and OR3 being 2.866 (2.203, 3.729), 1.680 (1.352, 2.086), and 1.707 (1.366, 2.132), respectively. Those results suggested a co-dominant model of gene effect on CAD (figure 3A and 3B). Several other possible genetic models were also estimated for references: dominant model, OR4 being 1.965 (1.594, 2.423); recessive model, OR5 being 1.971 (1.594, 2.438); additive model, OR6 being 1.689 (1.480, 1.927). No genetic model presented significant heterogeneity (table 4).

For IS, in subgroup analysis, pooling the 2 studies (n = 1717, 688 patients and 1029 controls) demonstrated crude OR1 of 0.684 (0.506, 0.926), OR2 of 1.216 (0.749, 1.974) and OR3 of 0.556 (0.341, 0.907) with marginal heterogeneity for OR2, evident heterogeneity for OR3 and no heterogeneity for OR1. Logistic regression showed that the overall gene effect was statistically significant, with the modified OR1, OR2, and OR3 being 0.666 (0.477, 0.930), 1.190 (0.866, 1.635), and 0.562 (0.402, 0.787), respectively. Those results suggested a recessive protective model of gene effect on IS (OR5 0.582 95% CI [0.393, 0.864]) (figure 2B). Several other possible genetic models were also estimated: co-dominant model, OR1 and OR2 being 0.666 (0.477, 0.930) and 1.190 (0.866, 1.635); dominant model, OR4 being 0.992 (0.691, 1.423); additive model, OR6 being 0.785 (0.679, 0.909). Recessive and co-dominant models showed significant heterogeneity (table 5).

**Figure 4:**
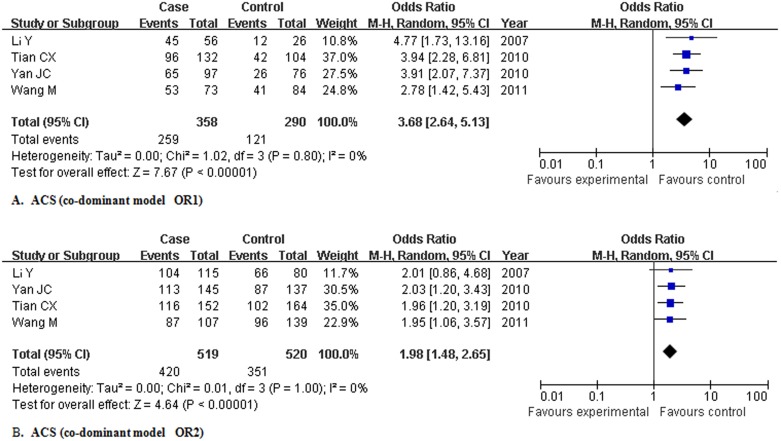
Meta-analysis for the association of rs1883832 with acute coronary syndrome in Chinese population. Abbreviation: ACS, acute coronary syndrome; OR, odds ratio; M-H, Mantel-Haenszel method.

For ACS, 4 studies (n = 1967, 1281 patients and 686 controls) were pooled to yield crude OR1 of 3.679 (2.637, 5.133), OR2 of 1.983 (1.485, 2.649) and OR3 of 1.852 (1.423, 2.409) without heterogeneity. Logistic regression showed that the overall gene effect was statistically significant, with the modified OR1, OR2, and OR3 being 3.674 (2.638, 5.116), 1.981 (1.483, 2.646), and 1.854 (1.428, 2.408), respectively. Those results suggested a co-dominant model of gene effect on ACS (figure 4A and 4B). Results under several other possible genetic models were also estimated: dominant model, OR4 being 2.421 (1.835, 3.193); recessive model, OR5 being 2.211 (1.719, 2.844); additive model, OR6 being 1.909 (1.619, 2.251). No genetic models showed evidence of significant heterogeneity (table 6).

**Table 6:** Meta-analysis for the association of rs1883832 with acute coronary syndrome.

Genetic model	OR 95%CI	P	P heterogeneity	I^2^
C/C versus T/T	Crude OR1: 3.679 (2.637, 5.133)	<0.001	0.796	0%
	Modified OR1: 3.674 (2.638, 5.116)	<0.001	–	–
C/T versus T/T	Crude OR2: 1.983 (1.485, 2.649)	<0.001	1	0%
	Modified OR2: 1.981 (1.483, 2.646)	<0.001	–	–
C/C versus C/T	Crude OR3: 1.852 (1.423, 2.409)	<0.001	0.642	0%
	Modified OR3: 1.854 (1.428, 2.408)	<0.001		–
Dominant model (C/C and C/T) versus T/T	OR4: 2.421 (1.835, 3.193)	<0.001	0.985	0%
Recessive model C/C versus (C/T and T/T)	OR5: 2.211 (1.719, 2.844)	<0.001	0.615	0%
Additive model	OR6: 1.909 (1.619, 2.251)	<0.001	0.714	0%

Included publications: Li Y, 2007; Tian CX, 2010; Yan JC, 2010; Wang M, 2011.

Likelihood ratio test: LR = 62.64, P<0.001.

Abbreviation: ACS, acute coronary syndrome; OR, odds ratio; 95%CI, 95% confidence interval.

### Sensitivity Study

Sensitivity study was conducted to assess the effects of each study on the pooled results of dominant model for atherosclerosis, co-dominant model for CAD and recessive model for IS, respectively. As showed in table 7, no single study, especially the one which does not observe HWE, significantly altered the pooled ORs with corresponding 95%CI, indicating the robustness of the current results. As well, the effects of each study on the pooled results of additive model for CAD and IS were evaluated, respectively. Results were similar before and after removal of each study (table 7).

**Table 7:** Sensitivity analysis.

Atherosclerosis		
Study omitted	Dominant model	Additive model
	Crude OR4 95%CI	Crude OR6 95%CI
Li Y, 2007	1.570(1.111,2.218)	1.275(0.905,1.796)
Wang CP, 2009	1.602(1.086,2.360)	1.324(0.911,1.926)
Tian CX, 2010	1.503(1.079,2.093)	1.274(0.908,1.787)
Yan JC, 2010	1.657(1.118,2.456)	1.353(0.924,1.981)
Wang M,2011	1.565(1.096,2.235)	1.324(0.918,1.909)
Ma Y, 2013	1.719(1.175,2.513)	1.511(1.078,2.119)
Zhang BK,2013	1.825(1.475,2.259)	1.493(1.082,2.060)
Combined	1.631(1.176,2.260)	1.361(0.981,1.889)
**IS**		
**Study omitted**	**Recessive model**	**Additive model**
	**Crude OR5 95%CI**	**Crude OR6 95%CI**
Ma Y, 2013	0.698(0.533,0.913)	0.810(0.677,0.968)
Zhang BK,2013	0.466(0.320,0.676)	0.737(0.571,0.951)
Combined	0.582(0.393,0.864)	0.785(0.678,0.909)
**CAD**		
**Study omitted**	**Co-dominant model**	**Additive model**
	**Crude OR1 95%CI Crude OR2 95%CI**	**Crude OR6 95%CI**
Li Y, 2007	2.762(2.095,3.643) 1.670(1.332,2.095)	1.654(1.443,1.896)
Wang CP, 2009	3.013(2.124,4.274) 1.721(1.315,2.253)	1.730(1.443,2.074)
Tian CX, 2010	2.600(1.915,3.529) 1.630(1.276,2.082)	1.607(1.383,1.868)
Yan JC, 2010	3.161(2.332,4.286) 1.804(1.398,2.327)	1.773(1.525,2.060)
Wang M,2011	2.913(2.093,4.056) 1.655(1.309,2.093)	1.711(1.444,2.028)
Combined	2.869(2.197,3.746) 1.691(1.359,2.105)	1.689(1.480,1.927)

Abbreviation: OR, odds ratio; 95%CI, 95% confidence interval; IS, is chemic stroke; CAD, coronary artery disease.

### Publication Bias

**Figure 5:**
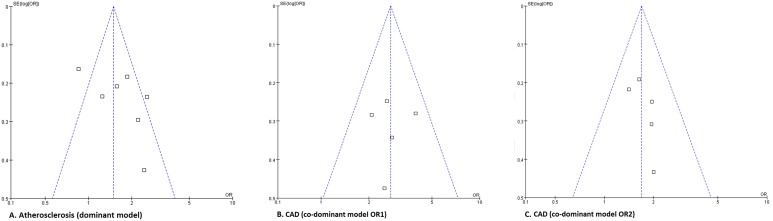
Funnel plots for the publication bias of atherosclerosis (A, dominant model) and coronary artery disease (B and C, co-dominant model). Abbreviation: CAD, coronary artery disease; OR, odds ratio.

The funnel plots and the Egger’s tests demonstrated that there was no publication bias in the dominant model for atherosclerosis (p for Egger’s test: p dominant model = 0.151; p additive model = 0.108) and co-dominant model and additive model for CAD (p for Egger’s test: p dominant model [OR1] = 0.397; p dominant model [OR2] = 0.253; p additive model = 0.379) (figure 5). Attempt has been made at identifying studies not in the published literature. However, we did not obtain any unpublished results.

## Discussion

In the current meta-analysis, pooling the data from 7 published case-control studies indicated a significant association between a single nucleotide polymorphism (SNP) rs1883832 in CD40 gene and atherosclerosis under dominant model in Chinese population. Subgroup analysis presented that C allele of rs1883832 conferred a higher risk for CAD or ACS under all genetic models, with co-dominant model potentially being the most appropriate model. However, a protective role of gene effect was noted for C allele in ischemic stroke (IS) under recessive and additive models. Our results demonstrated that rs1883832 contributed to an overall increased risk of atherosclerosis and provided clear evidence of distinct effect of rs1883832 on CAD (ACS) and IS in Chinese population, respectively. To our knowledge, this study was the first performed to pool published case-control studies to obtain estimates for association of rs1883832 with atherosclerosis in populations of Chinese ethnicity.

Genetic association studies with case-control design usually have a lack of power to replicate the associations from genome-wide association studies (GWAS). The difficulty of replication occurs partially due to the modest effect of those SNPs in complex diseases. Additionally, the overestimation of genetic effect may render replication studies underpowered and possibly fail because the necessary sample size is underestimated based on the original data from GWAS, a phenomenon known as the “winner’s curse” [19]. Our pooled results demonstrated a relatively high frequency of C allele in controls and large magnitudes of effect of C allele on atherosclerosis in Chinese. And our studies had an adequate sample size and 100% study power to evaluate the precise effect of rs1883832 on atherosclerosis in Chinese population. On the basis of the methods proposed by Thakkinstian et al. [10], [12], dominant model was probably the ideal mode of inheritance for atherosclerosis and suggested a positive association between C allele and an excess risk for atherosclerosis. Though between-study heterogeneity was obvious for dominant model, the following meta-regression analysis attributed the heterogeneity totally to disease category. Subsequent subgroup analysis for CAD and ACS suggested a potential co-dominant model as the finest mode. The dominant model showed an approximate 2-fold and 2.5-fold higher risk for CAD and ACS in individuals carrying at least one C allele than in ones with TT genotype, respectively. Furthermore, each additional C allele led to an average of nearly 1.7-fold and 1.9-fold increase in risk for CAD and ACS under additive model, respectively. Heterogeneity was completely removed for all estimates for CAD and ACS. However, subgroup analysis for IS presented an inverse outcome that C allele was associated with a reduction of nearly 40% risk for IS in 2 C allele carriers than in ones with CT and TT genotypes under recessive model, and each additional C allele lowered a 20% risk for IS on average under additive model. Heterogeneity in the subgroup analysis for IS was removed for additive model and reduced for recessive model. In our study, LR test was applied to gauge that the overall gene effect was statistically significant given an inflated type I error in the context of multiple tests. Further modified ORs were produced using logistic regression analysis to better estimate effect sizes and we found only subtle quantitative changes in modified ORs compared to respective crude ORs calculated by the traditional method. Due to the heterogeneity for atherosclerosis (overall and IS) and the inclusion of one study that did not observe HWE, sensitivity analysis was carried out to assess the stability of the estimates for the most appropriate genetic models for atherosclerosis (overall, CAD and IS, respectively) and additive models for CAD and IS. No single study markedly altered the estimates of the effect sizes and statistical differences remained significant before and after removal of each study, suggesting high robustness of our estimates. As well, we observed no publication bias in our meta-analysis.

**Figure 6:**
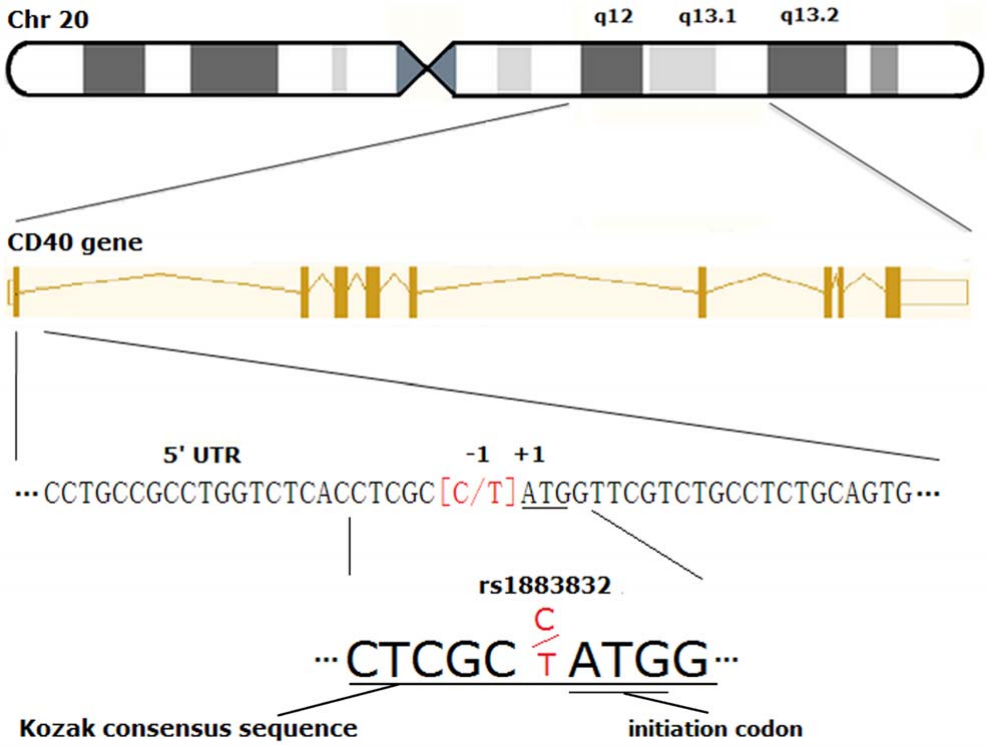
The specific position of rs1883832. Abbreviation: Chr, Chromosome; 5′ UTR, 5′ untranslated region.

CD40-CD40L interaction has recently been linked with the pathogenesis of atherosclerosis. Previous studies demonstrated the elevation of serum-soluble CD40L levels which may serve as one key indicator of an independent, increased risk of major adverse cardiovascular events in patients with unstable CAD. The expression of the CD40-CD40L system on platelets and B lymphocytes was as well found to be up-regulated in patients with ACS [15], [20]–[22]. In addition, soluble CD40L is potentially applied as a biomarker for atherosclerotic plague activity [14], [23]. The involvement of CD40-CD40L interaction in the inflammatory responses aggravates the development of atherosclerotic diseases and destabilization of plaques, which may ultimately renders vulnerable plagues susceptible to rupture and initiate lethal thrombosis and stenosis in ACS [24], [25]. The SNP rs1883832 maps in the functional region of the CD40 gene, i.e. the kozak consensus sequence (figure 6) and has recently been associated with ACS risk independent of the traditional risk factors such as hypertension and diabetes mellitus [7], [13]. Alteration of Kozak sequence which comprises 6 to 8 nucleotides before and after the initiation codon, may have an impact on the initiation of transcriptional activity and final gene expression [26], [27]. In Grave’s disease, up-regulation of CD40 by rs1883832 places an individual at an increased disease risk [28]. Similarly, C allele appeared to contribute to the over-expression of CD40 on not only platelets which are central to the formation of thrombosis in ACS patients, but also B lymphocytes and monocytes [7], [15], [17]. C allele is also positively associated with an increased risk of rupture of complex coronary atherosclerotic plagues underlying ACS [17]. Of note, CD40-CD40L interaction and rs1883832 may not promote the disease progression of stable angina (SA). Yan et al. [7]. reported no significant difference in C allele frequency or genotype distribution between the SA group and controls. The same study team demonstrated similar CD40-CD40L system expression levels of SA group and controls. Moreover, C allele frequency and genotype distribution in the group with stable plagues underlying SA was comparable with those of controls [17]. Thus rs1883832 is hypothesized to impact primarily on plague vulnerability rather than the initiation and development of atherosclerosis. We noted in our study the larger magnitudes for ACS risk than those for CAD risk, which might be consistent with the above assumption. However, C allele was negatively associated with the susceptibility to IS in our study. It might be a dissimilar genetic or etiologic contribution of rs1883832 to IS. It was indicated that altered CD40-CD40L interaction affected thrombus composition and its pattern of lyses. Another possibility was that aberrant CD40 isoforms might result in the changes in CD40-CD40L binding dynamics and tighter platelet-leukocyte adhesion, which increased IS risk [29]. Zhang et al. presented that TT genotype carriers showed higher CD40 expression and serum-soluble CD40 concentration in male IS patients [16]. Moreover, geographic variation and genetic heterogeneity may explain partially the different allele frequencies between CAD (ACS) and IS patients, and the high heterogeneity of allele frequencies in controls between different studies observed in our study. For genetic association studies, the same or different research groups occasionally utilize repetitive samples (especially controls). When a meta-analysis is conducted incorporating duplicate publications, a seemingly significant result may be obtained whereas the association is not robustly reproduced with a truly lower sample size. Besides, it may lead to a deviated estimate of the gene effects. Thus the potential duplicate-study effects were carefully examined and finally excluded in our analysis.

## Limitations

We wish to point out a few limitations of the present meta-analysis that readers should consider when interpreting the study results. First, inclusion of retrospective observational studies based on case-control design inevitably introduced a source of potential bias that inherently exists in non-randomized, unblinded design. Second, we failed to eliminate the heterogeneity in the subgroup analysis for IS. Third, atherosclerosis is a complex disorder triggered by multiple susceptibility genes and environmental factors. However, lacking of further evaluation of gene-gene and gene-environment interactions may have a diluting influence on the estimates for rs1883832. Fourth, other SNPs within or near CD40 gene, including rs1535045, rs3765459, rs4810485, rs3092952 and rs3092920, could be constructed statistically with rs1883832 to form multi-SNP haplotypes, which fine-map this region and are potentially more informative than individual SNP. Linkage disequilibrium (LD) pattern of this region should as well be determined using genotyping data of these SNPs [30]–[33]. It is likely that rs1883832 is a marker SNP in strong LD with the genuine causal loci. Thus the causal variants conferring an increased risk of atherosclerosis may remain unclearly determined. In addition, distinct LD patterns exist in different ethnicities whereas our meta-analysis did not analyze the association of rs1883832 with atherosclerosis in other populations. Although it might introduce less heterogeneity between the study populations, the restriction to Chinese ethnicity is a limitation in terms of the generalizability of our finding. Genetic association studies in other populations and multi-ethnic meta-analysis for this association are expected to be performed in near future. Fifth, only a portion of atherosclerotic diseases were investigated in our study. Thus risk of other diseases, such as peripheral or renal artery diseases and overall risk of atherosclerosis need to be further clarified.

## Conclusions

Our meta-analysis suggests that C allele rs1883832 in CD40 gene is positively associated with susceptibility to atherosclerosis, CAD and ACS whereas C allele appears to contribute to a decreased risk of IS in Chinese population. In the future, large scale case-control studies with rigorous design are called for in different ethnicities to replicate the association between rs1883832 and atherosclerosis and various atherosclerotic diseases. Further efforts for fine-mapping of CD40 gene region and functional analysis are required to identify causal variants and elucidate detailed mechanisms of CD40 polymorphisms in atherosclerosis.

## Supporting Information

Table S1 **Genetic models tested in this meta-analysis.** Abbreviation: OR, odds ratio.
(DOCX)

Checklist S1 **PRISMA checklist for this meta-analysis.**
(DOC)
