# Supplementary material for: The SNP rs1883832 in CD40 Gene and Risk of Atherosclerosis in Chinese Population: A Meta-Analysis
Source: PLoS One. 2014 May 14;9(5):e97289. doi: 10.1371/journal.pone.0097289 (PMC4020827; doi:10.1371/journal.pone.0097289)
Supplement: Table S1 — Genetic models tested in this meta-analysis. Abbreviation: OR, odds ratio. (DOCX) [file pone.0097289.s001.docx]

**Table S1**

| Genetic models |  |  |  | Definition and Model selection |
| --- | --- | --- | --- | --- |
| General model |  |  |  |  |
|  | TT | CT | CC | Mostly used in genetic association studies OR1 (C/C versus T/T), OR2 (C/T versus T/T), and OR3 (C/C versus C/T) |
| Cases | x1 | x2 | x3 |  |
| Controls | y1 | y2 | y3 |  |
| Dominant model |  |  |  |  |
|  | TT | CT + CC | | Allele C increases/decrease risk OR1 = OR2 ≠ 1 and OR3 = 1 |
| Cases | x1 | x2+x3 | |  |
| Controls | y1 | y2+y3 | |  |
| Recessive model |  |  |  |  |
|  | TT + CT | | CC | Two copies of allele C required for increased/decreased risk OR1 = OR3 ≠ 1 and OR2 = 1 |
| Cases | x1+x2 | | x3 |  |
| Controls | y1+y2 | | y3 |  |
| Co-dominant or Additive model |  |  |  |  |
|  | TT | CT | CC | Additive model: n-fold increased/deceased risk for CT, 2n increased/deceased risk for TT Co-dominant model: each additional C allele (not equally) increases or decreases risk, OR1 > OR2 > 1 and OR1 > OR3 > 1 (or OR1 < OR2 < 1 and OR1 < OR3 < 1) |
| Cases | x1 | x2 | x3 |  |
| Controls | y1 | y2 | y3 |  |

**Genetic models tested in this meta-analysis**

Abbreviation: OR, odds ratio
