# Supplementary material for: The SNP rs1883832 in CD40 Gene and Risk of Atherosclerosis in Chinese Population: A Meta-Analysis
Source: PLoS One. 2014 May 14;9(5):e97289. doi: 10.1371/journal.pone.0097289 (PMC4020827; doi:10.1371/journal.pone.0097289)
Supplement: Checklist S1 — PRISMA checklist for this meta-analysis. (DOC) [file pone.0097289.s002.doc]

| **Section/topic** | **#** | **Checklist item** | **Reported on page #** |
| --- | --- | --- | --- |
| **TITLE** | | |  |
| Title | 1 | The SNP rs1883832 in CD40 Gene and Risk of Atherosclerosis in Chinese Population: a meta-analysis | Title |
| **ABSTRACT** | | |  |
| Structured summary | 2 | **Background**: The complications of atherosclerosis such as coronary and cerebrovascular disease, are the most prevalent causes of mortality and morbidity worldwide. A single nucleotide polymorphism (SNP) rs1883832 (-1C/T) in CD40 gene has been recently suggested to contribute to the susceptibility to atherosclerosis in Chinese population; however, previous genetic association studies yielded inconsistent results.  **Methods**: A meta-analysis of eligible studies reporting the association between rs1883832 and atherosclerosis in Chinese population was carried out.  **Results**: Pooling 7 eligible case-control studies involving 2129 patients and 1895 controls demonstrated a significant association between rs1883832 and atherosclerosis under dominant model [odds ratio (OR)=1.631, 95% confidence interval [CI] [1.176, 2.260]] in Chinese population with evident heterogeneity. Meta-regression analysis indicated that the heterogeneity could be completely explained by disease category. In subgroup analysis, rs1883832 conferred ORs of 2.866 (C/C versus T/T, 95%CI [2.203, 3.729]) and 1.680 (C/T versus T/T, 95%CI [1.352, 2.086]) for coronary artery disease (CAD) under co-dominant model without heterogeneity. Similar results were obtained for acute coronary syndrome (ACS) (C/C versus T/T, 3.674, 95%CI [2.638, 5.116]; C/T versus T/T, 1.981, 95%CI [1.483, 2.646]). The other genetic models including dominant, recessive and additive models, yielded consistent results without heterogeneity for CAD and ACS, respectively. However, a protective role was found for C allele in ischemic stroke (IS) under recessive model (0.582, 95%CI [0.393, 0.864]) and additive model (0.785, 95%CI [0.679, 0.909]) with reduced heterogeneity.  **Conclusions:** This meta-analysis provided evidence of association of rs1883832 C allele with an overall increased risk of atherosclerosis but distinct effect of C allele on CAD (including ACS) and IS in Chinese population, respectively. | Abstract |
| **INTRODUCTION** | | |  |
| Rationale | 3 | Accumulating evidence has suggested a pivotal role of CD40-CD40L interaction in the pathogenesis of atherosclerosis. CD40 is a 50-kDa cell surface transmembrane glycoprotein receptor of the tumor necrosis factor receptor (TNFR) superfamily, which is expressed on the surface of immune cells as well as non-immune cells such as B cells, monocytes and platelets and determines T cell responses to antigen presentation and B cells immunoglobulin isotype switching. A C/T single nucleotide polymorphism (SNP) (rs1883832) in the 5’ untranslated region of CD40 gene located at the -1 position within the Kozak sequence has been recently associated with CD40 protein expression and susceptibility to atherosclerotic diseases in Chinese population. | Introduction |
| Objectives | 4 | We undertook the present meta-analysis of relevant literature to evaluate the association of rs1883832 (-1C/T) in CD40 gene with atherosclerosis (overall, CAD, acute coronary syndrome [ACS] and IS) in Chinese population, with the following objectives: A) to estimate C allele frequencies in controls; B) to assess if the effect of rs1883832 on atherosclerosis (overall, CAD, ACS and IS, respectively) is statistically significant in Chinese population; if so, C) to estimate the magnitude of effect of C allele and determine the most appropriate genetic model(s) for atherosclerosis (overall, CAD, ACS and IS, respectively) in Chinese population. | Introduction |
| **METHODS** | | |  |
| Protocol and registration | 5 | There is no review protocol and registration to be reported here. |  |
| Eligibility criteria | 6 | We sought eligible studies which met the following criteria: (1) published studies based on case-control design assessing the association between rs1883832 (CD40 gene -1C/T) and atherosclerosis; (2) involving subjects restricted to Chinese ethnicity; (3) providing sufficient data for calculating genotypic odds ratio (OR) with corresponding 95% confidence interval (95% CI). Non-English language studies were excluded, as were review articles, case reports, editorial comments and animal studies. It inevitably introduces a source of bias when a meta-analysis incorporates the genetic association studies from the same or different research group(s) utilizing duplicate samples. Thereby, it is obligatory to exclude in advance those repetitive publications to avoid the duplicate-study effects on our meta-analysis. The recruiting periods and hospitals were checked thoroughly for all the included studies. Additionally, the corresponding authors were contacted directly via email to confirm whether or not there were duplicate results to be eliminated. | Materials and methods |
| Information sources | 7 | MEDLINE and EMBASE were independently searched by two reviewers for potentially eligible studies from the earliest available date to August 2013. | Materials and methods |
| Search | 8 | The search terms used either alone or in combination included “rs1883832 -1C/T, CD40, TNFRSF5, atherosclerosis, coronary artery disease, acute coronary syndrome, ischemic stroke and cerebral infarction”. Reference lists from retrieved articles were also manually searched for articles meeting our criteria. | Materials and methods |
| Study selection | 9 | Abstracts identified using our search strategies were reviewed separately by two reviewers (Xiaochun Ma and Yan Yun). The full-text articles that potentially met criteria were then reviewed in duplicate to determine the inclusion in the analysis. Then all the authors further critically reviewed the studies upon which the 2 reviewers disagreed. Either the inclusion or exclusion of a certain study was agreed upon by all the authors finally. | Materials and methods |
| Data collection process | 10 | Data extraction was performed independently by two reviewers (Xiaochun Ma and Chi Ma). In the process of data abstraction, the two authors were blinded to the information of each research group conducting the study. | Materials and methods |
| Data items | 11 | Extracted data included: (1) author’s name, year of publication and journal; and (2) study design, sample size, characteristics of cases and controls (geographic location or ethnicity of study population, control source, male/female rate and mean age), genotyping method, distribution of genotypes and alleles in cases and controls. | Materials and methods |
| Risk of bias in individual studies | 12 | The quality of studies was assessed with the Newcastle-Ottawa scale (NOS) for observational studies8. Eight items, in each of which a series of response options is provided, are categorized into three perspectives in NOS: four for the Selection of the study group (S), one for the Comparability (C) of the groups and three for Exposure of interest (E). A ‘star system’ is utilized for the assessment of study quality, such that a study can be awarded a maximum of one star for each numbered item within the Selection and Exposure categories while a maximum of two stars can be assigned for Comparability. The NOS scores from zero to nine stars9. | Materials and methods |
| Summary measures | 13 | Odds ratio (ORs) and corresponding 95% confidence intervals (CI) | Materials and methods |
| Synthesis of results | 14 | Hardy-Weinberg equilibrium was assessed again for each study by goodness-of-fit x2 test (P＞0.05) only in control groups. Pooled frequency of the A allele was estimated using the inverse variance method previously described by Thakkinstian et al10 in which random-effects or fixed-effects model was applied, depending on whether or not there is evident heterogeneity for allele frequencies across studies. Random-effects model introduces a ‘weight term’ that accounts for between-study variation and weight adjustment. Three crude ORs with corresponding 95% CIs, i.e. crude OR1 (C/C versus T/T), crude OR2 (C/T versus T/T) and crude OR3 (C/C versus C/T) were calculated for rs1883832. Cochran’s Q statistic and the I2 metric for heterogeneity were also estimated separately for crude OR1, OR2 and OR3. Three modified ORs and various genetic models would be measured afterwards only if there was no evident heterogeneity. For the Q statistic, a p value of less than 0.1 was considered statistically significant. For the I2 metric, I2＞50% indicates significant heterogeneity11. If heterogeneity was evident for at least one of the 3 crude ORs, a meta-regression model was employed to explore possible sources of heterogeneity across studies. Subgroup analysis was then conducted on the basis of the results of meta-regression analysis. If there was no heterogeneity, logistic regression analysis was performed for determining the gene effect, which protected against inflated type I error in the context of multiple tests. The overall gene effect was evaluated using likelihood ratio (LR) test. If the main effect of the genotype was statistically significant, three modified ORs, i.e. OR1 (C/C versus T/T), OR2 (C/T versus T/T), and OR3 (C/C versus C/T) were further calculated using logistic regression model. Three pairs of crude and modified ORs were then utilized to suggest the most appropriate or ideal genetic model previously described by Thakkinstian et al10,12 as follows:  If OR1 = OR3 ≠ 1 and OR2 = 1, then a recessive model is suggested.  If OR1 = OR2 ≠ 1 and OR3 = 1, then a dominant model is suggested.  If OR1 > OR2 > 1 and OR1 > OR3 > 1 (or OR1 < OR2 < 1 and OR1 < OR3 < 1), then a co-dominant model is suggested.  Besides the finest genetic model, several other possible genetic models were also assessed: co-dominant model (modified OR1 and OR2), (C/C versus T/T) and (C/T versus T/T); dominant model (OR4), (C/C and C/T) versus T/T; recessive model, C/C versus (C/T and T/T) (OR5); additive model (OR6) (table S1). For additive model, scores of 0, 1, and 2 were assigned to genotype CC, CT, and TT respectively and per-allele ORs were calculated by logistic regression model. Sensitivity analysis was performed to assess the effects of each study (especially the studies which do not observe HWE), on the pooled results. Publication bias was assessed using the Funnel plot method with Egger’s test. Random-effects models were chosen for pooling data and a p value of less than 0.05 was considered statistically significant for all estimates except for Q tests of heterogeneity. Agreement assessment between reviewers was evaluated using the Cohen k statistic. Stata 12.0 and Reviewer Manager 5.1 were utilized for performing the meta-analysis. | Materials and methods |

Page 1 of 2

| **Section/topic** | **#** | **Checklist item** | **Reported on page #** |
| --- | --- | --- | --- |
| Risk of bias across studies | 15 | Publication bias was assessed using the Funnel plot method with Egger’s test. Random-effects models were chosen for pooling the results from included studies | Materials and methods |
| Additional analyses | 16 | We have performed sensitivity study, subgroup study, likelihood ration test, logistic regression analysis and meta-regression analysis. Please see 14. | Materials and methods |
| **RESULTS** | | |  |
| Study selection | 17 | A total of 156 potential eligible citations were identified with the literature search, among which there were no articles written and published in languages other than English and Chinese. The titles of these citations were reviewed and 125 were rejected leaving 31 potentially eligible studies. After review of abstracts, an additional 14 were rejected. Seventeen studies were then retrieved in full-text to determine the inclusion. Assessment agreement of study selection between the two reviewers led to a k score of 0.84 (95% CI [0.79, 0.89]). Then a further discussion was conducted among all the authors with regard to the studies upon which the 2 reviewers disagreed. Finally, corporate critical review of full-text excluded 4 studies for not reporting Chinese population, 3 studies for being reviews and editorial comments, 3 studies for without data for rs1883832 (figure 1). All the included studies in this meta-analysis were written and published in English. Furthermore, there is currently no genetic association study concerning rs1883832 in atherosclerosis in other ethnicities, based on our search result. | Results |
| Study characteristics | 18 | We have summarized specifically this part in table1 and table2. Please see these 2 tables. | Results |
| Risk of bias within studies | 19 | All studies gained a score of S3C1E3 based on the Newcastle-Ottawa scale. (Selection=S; Comparability= C; Exposure=E) | Results |
| Results of individual studies | 20 | We have summarized specifically this part in table 2-6. Please see these tables. | Results |
| Synthesis of results | 21 | We have summarized specifically this part in table 3-7. Please see these tables. | Results |
| Risk of bias across studies | 22 | The funnel plots and the Egger’s tests demonstrated that there was no publication bias in the dominant model for atherosclerosis (p for Egger’s test: p dominant model=0.151; p additive model=0.108) and co-dominant model and additive model for CAD (p for Egger’s test: p dominant model [OR1]=0.397; p dominant model [OR2]=0.253; p additive model=0.379) (figure 5). Attempt has been made at identifying studies not in the published literature. However, we did not obtain any unpublished results. | Results |
| Additional analysis | 23 | We have summarized specifically this part in table 2-6. Please see these tables. | Results |
| **DISCUSSION** | | |  |
| Summary of evidence | 24 | In the current meta-analysis, pooling the data from 7 published case-control studies indicated a significant association between a single nucleotide polymorphism (SNP) rs1883832 in CD40 gene and atherosclerosis under dominant model in Chinese population. Subgroup analysis presented that C allele of rs1883832 conferred a higher risk for CAD or ACS under all genetic models, with co-dominant model potentially being the most appropriate model. However, a protective role of gene effect was noted for C allele in ischemic stroke (IS) under recessive and additive models. Our results demonstrated that rs1883832 contributed to an overall increased risk of atherosclerosis and provided clear evidence of distinct effect of rs1883832 on CAD (ACS) and IS in Chinese population, respectively. | Discussion |
| Limitations | 25 | We wish to point out a few limitations of the present meta-analysis that readers should consider when interpreting the study results. First, inclusion of retrospective observational studies based on case-control design inevitably introduced a source of potential bias that inherently exists in non-randomized, unblinded design. Second, we failed to eliminate the heterogeneity in the subgroup analysis for IS. Third, atherosclerosis is a complex disorder triggered by multiple susceptibility genes and environmental factors. However, lacking of further evaluation of gene-gene and gene-environment interactions may have a diluting influence on the estimates for rs1883832. Fourth, other SNPs within or near CD40 gene, including rs1535045, rs3765459, rs4810485, rs3092952 and rs3092920, could be constructed statistically with rs1883832 to form multi-SNP haplotypes, which fine-map this region and are potentially more informative than individual SNP. Linkage disequilibrium (LD) pattern of this region should as well be determined using genotyping data of these SNPs30-33. It is likely that rs1883832 is a marker SNP in strong LD with the genuine causal loci. Thus the causal variants conferring an increased risk of atherosclerosis may remain unclearly determined. In addition, distinct LD patterns exist in different ethnicities whereas our meta-analysis did not analyze the association of rs1883832 with atherosclerosis in other populations. Although it might introduce less heterogeneity between the study populations, the restriction to Chinese ethnicity is a limitation in terms of the generalizability of our finding. Genetic association studies in other populations and multi-ethnic meta-analysis for this association are expected to be performed in near future. Fifth, only a portion of atherosclerotic diseases were investigated in our study. Thus risk of other diseases, such as peripheral or renal artery diseases and overall risk of atherosclerosis need to be further clarified. | Discussion |
| Conclusions | 26 | In conclusion, our meta-analysis suggests that C allele rs1883832 in CD40 gene is positively associated with susceptibility to atherosclerosis, CAD and ACS whereas C allele appears to contribute to a decreased risk of IS in Chinese population. In the future, large scale case-control studies with rigorous design are called for in different ethnicities to replicate the association between rs1883832 and atherosclerosis and various atherosclerotic diseases. Further efforts for fine-mapping of CD40 gene region and functional analysis are required to identify causal variants and elucidate detailed mechanisms of CD40 polymorphisms in atherosclerosis. | Conclusions |
| **FUNDING** | | |  |
| Funding | 27 | We had no funding support for this meta-analysis. |  |

*From:*  Moher D, Liberati A, Tetzlaff J, Altman DG, The PRISMA Group (2009). Preferred Reporting Items for Systematic Reviews and Meta-Analyses: The PRISMA Statement. PLoS Med 6(6): e1000097. doi:10.1371/journal.pmed1000097

For more information, visit: **www.prisma-statement.org**.

Page 2 of 2
